# Interleukin-17A Drives IL-19 and IL-24 Expression in Skin Stromal Cells Regulating Keratinocyte Proliferation

**DOI:** 10.3389/fimmu.2021.719562

**Published:** 2021-09-20

**Authors:** Xiaofei Xu, Errol Prens, Edwin Florencia, Pieter Leenen, Luis Boon, Patrick Asmawidjaja, Anne-Marie Mus, Erik Lubberts

**Affiliations:** ^1^ Department of Rheumatology, Erasmus Medical Center, Rotterdam, Netherlands; ^2^ Department of Dermatology, Erasmus Medical Center, Rotterdam, Netherlands; ^3^ Department of Immunology, Erasmus Medical Center, Rotterdam, Netherlands; ^4^ Polypharma Biologics, Utrecht, Netherlands

**Keywords:** psoriasis, cytokines, skin, inflammation, IL-17A, Th17

## Abstract

IL-17A has been shown to be up-regulated in psoriasis lesions and is central to psoriasis pathogenesis. IL-19, along with other IL-20 subfamily cytokines such as IL-20 and IL-24, is induced by IL-17A and contributes especially to epidermal hyperplasia in psoriasis. However, the regulation, cellular sources of IL-19 and whether targeting of IL-17A by biologics influence IL-19 expression is not completely understood. To investigate the regulation of IL-19 by IL-17A in psoriasis, the imiquimod-induced psoriasis mouse (IMQ) model was used. Enhanced expression of IL-17A in the IMQ model was achieved by anti-IL-10 antibody treatment. Assessments of skin inflammation macroscopically, by histology and flow cytometry, all confirmed increased psoriatic symptoms. Interestingly, depletion of IL-10 markedly upregulated IL-23/IL-17 pathway related cytokines followed by a significant increase in IL-19 and IL-24. The up-regulation of IL-19 and IL-24, but not IL-17A, coincided with increased keratinocyte proliferation. To investigate the cellular source and effects of biologics on IL-19, human skin fibroblasts from healthy controls and psoriasis patients were cultured alone or co-cultured with activated memory CD4+ T cells. Besides IL-1β, IL-17A induced direct expression of IL-19 and IL-24 in skin fibroblasts and keratinocytes. Importantly, intrinsic higher expression of IL-19 in psoriatic skin fibroblasts was observed in comparison to healthy skin fibroblasts. Neutralization of IL-17A in the human skin fibroblast-T cell co-culture system significantly suppressed IL-19 and IL-24 expression. Together, our data show that IL-17A-induced IL-19 and IL-24 expression in skin stromal cells contribute to keratinocyte proliferation.

## Introduction

Psoriasis is a chronic autoimmune skin disease affecting around 2 to 3% of the Western population (1, 2). Psoriasis vulgaris, the plaque-forming phenotype, is the most common type and accounts for 85-90% of all patients with psoriasis (1, 3).

Aberrant regulation of pro-inflammatory and anti-inflammatory cytokines is considered important in the pathogenesis of psoriasis (1–4). Increased levels of IL-17A and elevated percentages of IL-17A-producing lymphocytes are found in psoriatic plaques (5–9). IL-17A alone, and in combination with other inflammatory cytokines such as TNFα, stimulate keratinocyte activation, proliferation, amplifies the immune response and perpetuates cell infiltration (10, 11). Biologics targeting IL-17A alone or in combination with IL-17F or the IL-17 receptor A (IL-17RA) are efficacious in the treatment of plaque psoriasis, highlighting the central role of the IL-17A pathway in psoriasis pathogenesis (12). Interleukin-19, a member of the IL-20 subfamily together with IL-20 and IL-24, is up-regulated in psoriatic lesions and contributes to keratinocytes hyperplasia in psoriasis (13). IL-19 is reportedly down-stream of the IL-23/IL-17 cascade and autocrine production of IL-19 by keratinocytes is shown to elicit keratinocyte proliferation (14). Recent data show that serum IL-19 levels reflect clinical improvement induced by anti-IL-17 biologic treatment (15). However, whether other resident cells, such as skin fibroblasts, are a source of IL-19 and whether *in situ* IL-19 expression is normalized in patients with psoriasis treated with anti-IL-17 therapy is not clear.

Previously, our group established a psoriasis-like skin inflammation model in mice using topical application of imiquimod (IMQ) (16). This model successfully re-captures most critical features of acute plaque formation in psoriasis such as keratinocyte hyper proliferation, acanthosis and parakeratosis (16). Like in human psoriasis, enhanced activity of the IL-23/IL-17 pathway was also involved in the IMQ-induced psoriasis mouse model (16). However, in contrast to the chronic natural course in human psoriasis, this mouse model does not develop into a chronic state of psoriasis, because of stabilization and even improvement of skin inflammation after 5 to 6 days. Interestingly, a clinical study in psoriasis patients showed that, non-lesional skin treated with IMQ initially developed typical features of psoriasis such as acanthosis and parakeratosis (17). Nevertheless, both clinical and histological features subsided thereafter and in this human model of IMQ-induced psoriasis, the induced lesions showed spontaneous improvement after 5 to 6 days. This improvement was accompanied by significantly lower expression of IL-17A and with a higher expression of IL-10 (17). This suggests that upregulation of IL-10 is involved in the spontaneous improvement of psoriasis symptoms after 5 to 6 days in murine IMQ model and probably explains the spontaneous improvement observed in the IMQ mouse model. Therefore, we used an anti-IL-10 antibody to investigate whether we could achieve enhanced expression of IL-17 in the IMQ-induced psoriasis mouse model and the accompanying visible psoriatic symptoms beyond day 5. *In vitro* assays with human skin fibroblasts from patients with psoriasis and healthy skin were performed to evaluate the direct induction of IL-19 by IL-17. In addition, an ex vivo human psoriasis skin co-culture system was used to examine the effects of biologics targeting IL-17A on IL-19 expression.

## Material and Methods

### IMQ-Induced Psoriasis Mouse Model

BALB/c mice (8-11 week-old) received daily topical application of 62.5mg 5% Aldara (3M Pharmaceuticals) on their shaved back skin. Control mice (n=6, pooled from two independent experiments) were treated with a thin layer of petrolatum (Fagron). Daily evaluation of the local psoriasis area and severity index (PASI) has been described previously (16). Every other day, 20 mg/kg body weight of anti-IL-10 or isotype control antibody (n=10 each, pooled from two independent experiments) was intraperitoneally (i.p.) injected, or 5 mg/kg body weight of dexamethasone (n=7, pooled from two independent experiments) was subcutaneously (s.c.) injected as an anti-inflammatory gold standard. Five and ten days after IMQ induction, mice were sacrificed for analysis. Food and water were provided ad libitum, and mice were kept under specific pathogen-free conditions. All experiments were approved by the Erasmus MC Dutch Animal Ethics Committee (DEC).

### Histology and Immunohistochemistry

After sacrifice, skin biopsies were taken and snap-frozen in TissueTek (Bayer). Sections were cut with a Leica cryostat. Gr-1 antibody (clone RB6-8C5) and Ki-67 antibody (Dako, A0047) were used for IHC staining. Subsequent steps were performed as described earlier (16).

Images were analyzed with LAX V4.12 program (Leica microsystems) or NDP view2 (Hamamatsu photonics). To measure epidermal thickness, the average of four measurements was used as the representative thickness per sample. To reduce variance between different experiments, thickness ratios were calculated. Specifically, each skin thickness was divided by the mean skin thickness of the isotype group from that experiment, and thereby mean values of thickness for isotype groups were always set at one.

### Flow Cytometry

Back skin (ca. 1 cm2) was digested in 50 μg/mL Liberase (Roche) at 4°C overnight and then at 37°C for 1 hour to create a single cell suspension and cells were stained with the following antibodies: CD45-BV785 (Biolegend, clone 104), CD11b-eF450 (eBioscience, clone M1/70), Ly6C-APC-Cy7 (BD pharmingen, clone AL-21) and Ly6G-PE-CF594 (BD horizon, clone 1A8). Samples were analyzed with LSR II flow cytometer (BD Biosciences) and results processed with FlowJo software (TreeStar).

Healthy peripheral blood mononuclear cells (PBMC) were obtained from buffy coats (Sanquin, Amsterdam, the Netherlands) and harvested with Ficoll density gradient centrifugation. For the co-culture experiments, memory T cells (CD4+CD45RO+CD14-CD25low/int) were sorted using the FACSAria cell sorter (BD Biosciences) and co-cultured with skin fibroblasts. Cells were stained with the following antibodies: CD14-APC-H7 (clone MϕP9), CD45RO-PerCP-Cy5.5 (clone UCHL1), both from BD Biosciences, and CD4-FITC (clone RPA-T4), CD25-PE Cy7 (clone BC96), both from Sony Biotechnology. The obtained cell purity was ≥ 98%.

### Reverse Transcription and Real-Time Polymerase Chain Reaction

RNA was isolated with TRIzol (Thermo Fisher) or Total RNA Miniprep Kit (Sigma-Aldrich). cDNA was synthesized with Superscript II after DNase treatment (both from Invitrogen). RT-PCR was performed with ViiA7 sequence detection system (Life Technologies). Gene expression of IL-17A, IL-17F, IL-19, IL-22, IL-23p19, IL-24 were normalized to glyceraldehyde 3-phosphate dehydrogenase (GAPDH) in mouse samples and hypoxanthine-guanine phosphoribosyltransferase (HPRT) in human samples. Primer sequences were summarized in **Supplementary Table S1**.

### Human Primary Skin Fibroblast Cultures and Co-Cultures With T Cells

Fibroblasts (n=6) from lesional skin of psoriasis patients were obtained from our biobank collection. Healthy skin samples (n=6) were obtained from healthy individuals who underwent cosmetic surgeries in the Sint Franciscus Hospital (Rotterdam, The Netherlands). Signed consents were provided by all healthy participants. Human primary skin fibroblasts were cultured from above skin samples as described previously (18). Passages 3-8 fibroblasts were seeded 1.0×10^4^ per well in 96-well culture plates and stimulated with IL-1β (0.01 ng/mL, 201-LB), TNFα (5 ng/mL, 210-TA), IL-17A (50 ng/mL, 317-IL), and IL-17F (500 ng/mL, 1335-IL) (all from R&D systems) for 24 hours (hrs).

In co-culture experiments, skin fibroblasts (1.0x10^4^) were co-cultured with CD4+CD45RO+CD14-CD25low/int T memory cells (2.5×10^4^) sorted from buffy coats (n=6). Soluble anti-CD3 and anti-CD28 (both from Sanquin, Amsterdam, The Netherlands) were added for 72 hrs. In addition, 100 μg/ml anti-IL-17A antibody (secukinumab, Novartis), 1μg/ml anti-TNF antibody (adalimumab, AbbVie), and an isotype IgG1κ antibody (Sigma-Aldrich) were used.

### Enzyme-Linked Immunosorbent Assay

Human IL-8 and IL-19 in culture supernatants was measured with ELISA (Invitrogen) and ELISA Duoset (R&D systems) following manufacturers’ instructions.

### Data Set Analysis

Public microarray data (GSE13355) were analyzed to compare mRNA expression of psoriatic lesional (n=64), non-lesional skin (n=58) and skin from healthy controls (n=58). Based on GSE13355, values of 216876_s_at (IL-17A), 220745_at (IL-19), 206569_at (IL-24) and 212021_s_at (MKI67) from gene expression profile GDS4602 were plotted. Dataset GSE53552 was analyzed for mRNA expression of psoriatic skin lesions following treatment with brodalumab (AstraZeneca). Psoriatic non-lesional (n=23), lesional (n=25), and day 8 (n=4), day 15 (n=19), day 43 (n=16) after treatments were included. 220745_at (IL-19), 206569_at (IL-24), 212022_s_at (MKI67) values from GDS5420 were plotted based on GSE53552.

### Statistics

Statistical differences were determined with paired or unpaired student’s *t* test. All data analyses were performed with GraphPad Prism V5 and P-values <0.05 were considered as significant.

## Results

### IL-10 Neutralization Enhances Skin Thickness and Scaling in the IMQ-Induced Psoriasis Mouse Model

**Figure 1:**
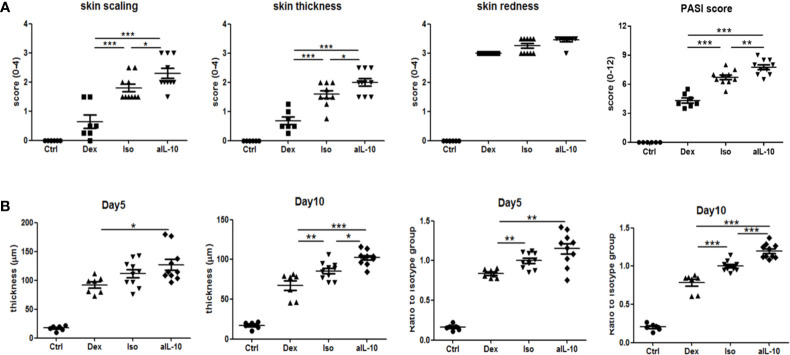
IL-10 neutralization worsens psoriatic symptoms and epidermal thickness in the IMQ-induced psoriasis mouse model. **(A)** At day 10, scores for skin scaling, thickness, redness and PASI in various groups following IMQ treatment. **(B)** Measured average epidermal thickness in psoriasis-like skin at days 5 and 10, and thickness ratios compared to isotype antibody controls at days 5 and 10. Data are shown as means ± SEMs. *P < 0.05, **P < 0.01, and ***P < 0.001.

The design of the IL-10 neutralization experiments is summarized in **Supplementary Figures S1A, B**. Local psoriasis area and severity index (PASI) score was used to evaluate psoriasis symptoms including skin scaling, thickness and redness. As shown in **Figure 1A**, ten days after IMQ treatment, macroscopic scores of skin scaling and thickness were significantly higher in the anti-IL-10 treated group (anti-IL-10) compared to the isotype antibody control group (isotype). This resulted in a significant higher PASI score after neutralizing IL-10 compared to the isotype control (**Figure 1A**). Dexamethasone treatment significantly improved both symptoms compared to either the anti-IL-10 or isotype group. No significant difference for skin redness was observed among groups. Details of kinetic data of the macroscopic scores were summarized in **Supplementary Figure S2A**. These data indicate that IL-10 neutralization enhances skin thickness and scaling in the IMQ-induced psoriasis mouse model beyond day 5.

### IL-10 Neutralization Increases Epidermal Thickness and Keratinocyte Proliferation in the IMQ-Induced Psoriasis Mouse Model

**Figure 2:**
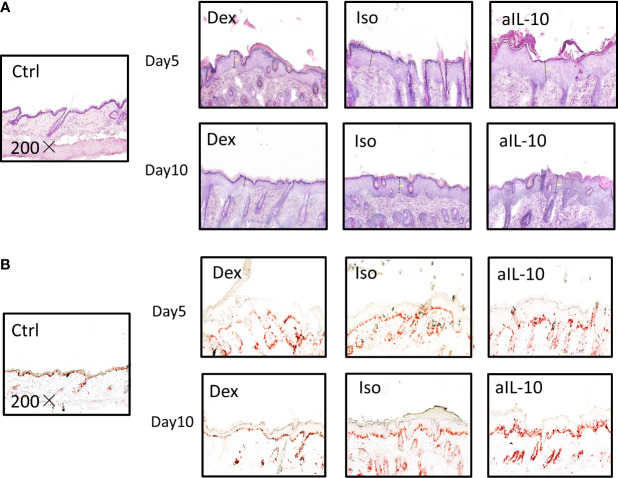
IL-10 neutralization increases keratinocyte proliferation and neutrophil accumulation in the IMQ-induced psoriasis mouse model. **(A)** Representative H&E staining results of skin sections in IMQ-induced psoriasis mouse model at days 5 and 10. **(B)** Representative IHC staining of Ki-67+ proliferating keratinocytes in skin sections of IMQ-induced psoriasis mouse model at days 5 and 10. All images were taken with 200× magnification.

To confirm the increased skin thickness observed macroscopically after anti-IL-10 treatment, H&E staining was performed and epidermal thickness was measured microscopically (**Figure 2A**). At day 5, epidermal thickness was significantly higher in the anti-IL10 compared to dexamethasone treatment (**Figure 1B**). At day 10, anti-IL-10 significantly increased epidermal thickness compared to the isotype group, whereas dexamethasone significantly reduced this compared to both groups (**Figure 1B**). This is in line with our macroscopic findings of increased skin thickness with the PASI score in the anti-IL-10 treated group (**Figure 1A**).

As indicated before, compared to isotype group, ratios of epidermal thickness in the anti-IL-10 treated group were slightly increased at day 5, but significantly increased at day 10 (**Figure 1B**). Both epidermal thickness and thickness ratios in the isotype group were comparable to saline-treated IMQ groups (**Supplementary Figure S2B**). Ki-67 staining was performed to further identify proliferating cells. As shown in **Figure 2B**, 1-2 layers of keratinocytes in the epidermal stratum basale were Ki-67+ in dexamethasone group, 2-3 layers were Ki-67+ in isotype group, while 4-5 layers were Ki-67+ in anti-IL-10 group (at days 5 and 10). This is in line with the increased thickness of the epidermis in the anti-IL-10 treated group at day 10 (**Figure 1B**). Taken together, our data indicate that in the IMQ-induced psoriasis mouse model, IL-10 neutralization enhances skin thickness through facilitating keratinocyte proliferation.

### IL-10 Neutralization Increases the Recruitment of Neutrophils and Monocytes Into the Skin

**Figure 3:**
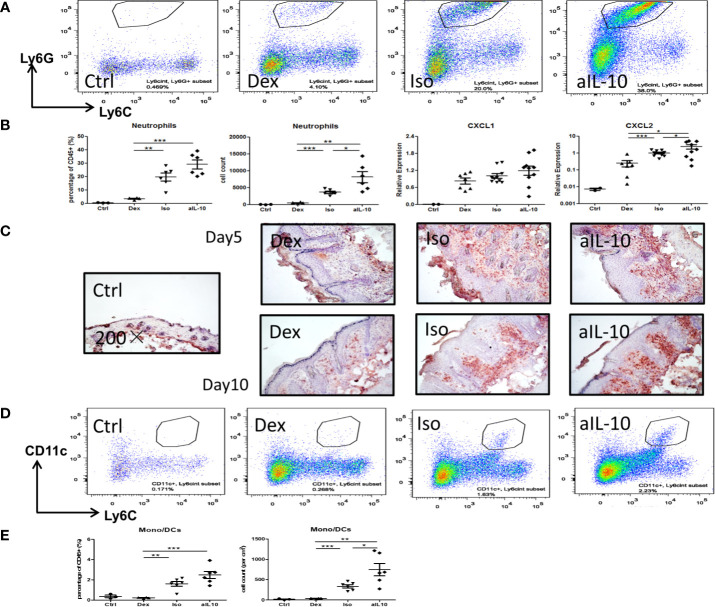
IL-10 neutralization increases neutrophil and monocyte/DC infiltration in the IMQ-induced psoriasis mouse model. **(A)** Flow cytometry staining of Ly6G+Ly6Cint neutrophils among pregated CD45+CD11b+ cells in lesional psoriasis-like skin at day10. **(B)** Percentages and cell numbers of neutrophils among total CD45+ immune cells at day 10 in flow cytometry analysis and levels of the neutrophil chemokines, CXCL1 and CXCL2, in lesional psoriasis-like skin. **(C)** Representative IHC staining of Gr-1+ neutrophils in skin sections of the IMQ-induced psoriasis mouse model at days 5 and 10. **(D)** Flow cytometry staining of CD11c+Ly6Cint monocyte-derived dendritic cells in lesional psoriasis-like skin at day10. **(E)** Percentages and cell numbers of monocyte-derived dendritic cells (mono/DCs) among total CD45+ immune cells at day 10 using flow cytometry. Data are shown as means ± SEMs. *P < 0.05, **P < 0.01, and ***P < 0.001.

Flow cytometry showed representative staining results of infiltrating neutrophils (CD11b+Ly6CintLy6G+) in lesional skin at day 10 (**Figure 3A**). Both neutrophil numbers and percentages were increased after neutralization of IL-10 compared to the isotype group (**Figure 3B**). Treatment with dexamethasone significantly reduced both parameters compared to the other groups (**Figure 3B**). At days 5 and 10, IHC staining confirmed the enhanced recruitment of Gr-1+ neutrophils in IMQ lesional skin in the anti-IL-10 treated group compared to the isotype group (**Figure 3C**). Neutrophil chemokine CXCL2, but not CXCL1, was upregulated after neutralizing IL-10 and correlated with enhanced neutrophil recruitment (**Figure 3B**). Similar results were found for CD11c+Ly6Cint monocytes-derived dendritic cells (mono/DCs) (**Figures 3D, E**). The isotype group showed similar cell numbers and percentages of neutrophils and mono/DCs compared to the saline-treated IMQ group (**Supplementary Figures S2C, D**), indicating that no specific immune effects were induced by the isotype antibody injections. These data indicate that IL-10 neutralization in the IMQ-induced psoriasis mouse model results in enhanced persistent inflammation, as evidenced by enhanced influx of neutrophils and mono/DCs into lesional skin.

### IL-10 Neutralization in the IMQ-Induced Psoriasis Mouse Model Results in an Early Upregulation of the IL-23/IL-17 Immune Pathway Related Cytokines Followed by a Subsequent Later Increase of IL-19 and IL-24 in the Skin

**Figure 4:**
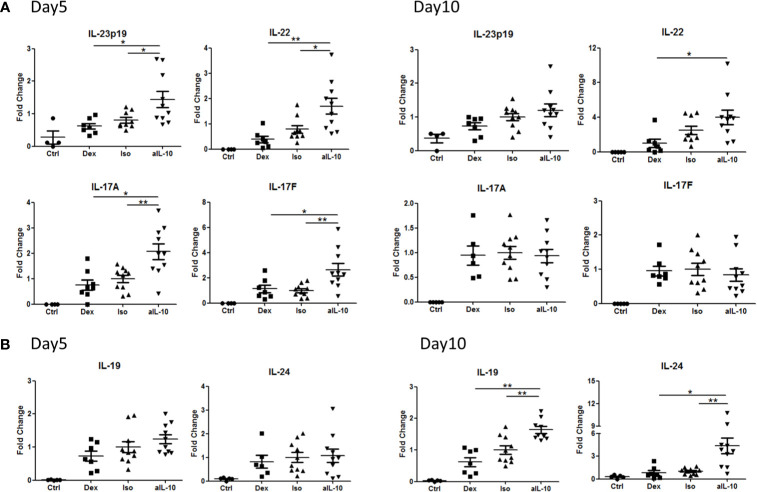
Early and late up-regulation of IL-23/IL-17 cytokines and IL-20 subfamily cytokines in the skin during anti-IL-10 treatment in IMQ-induced psoriasis mouse model. **(A)** At days 5 and 10, mRNA expression of IL-23, IL-22, IL-17A and IL-17F in various groups following IMQ treatment. **(B)** At days 5 and 10, mRNA expression of IL-19 and IL-24 in various groups following IMQ treatment. Data are shown as means ± SEMs. *P < 0.05, **P < 0.01.

In psoriasis, IL-17-producing T helper cells (Th17) are central in the pathogenesis and Th17-related cytokines such as IL-17A, IL-17F and IL-22, together with TNFα, drive epidermal hyperplasia (9, 16, 19). Therefore, we investigated the effects of IL-10 neutralization on T cells and T cell cytokine expression in the IL-23/IL-17-dependent IMQ-induced psoriasis mouse model (16). Neutralizing IL-10 did not change the number of CD3+ T cells, including CD4+ and γδ T cells at day 10 (**Supplementary Figures S3A–C**). However, at day 5, IL-23p19, IL-22, IL-17A and IL-17F were significantly increased in the anti-IL-10 treated group compared to the isotype control group (**Figure 4A**). Interestingly, no difference in expression of these cytokines was found between these two groups at day 10 (**Figure 4A**). In contrast, the IL-20 subfamily cytokines, IL-19 and IL-24, were significantly upregulated only at day 10 but not at day 5 (**Figure 4B**) which correlated with the significant increase of epidermal thickness at day 10. This indicates that IL-19 and IL-24, rather than IL-22, IL-17A or IL-17F, were responsible for the late stage (day 10) keratinocyte hyper-proliferation and acanthosis in the IMQ-induced psoriasis mouse model during anti-IL-10 treatment.

### IL-17 Induces Human Skin Fibroblasts to Produce IL-19 and IL-24

**Figure 5:**
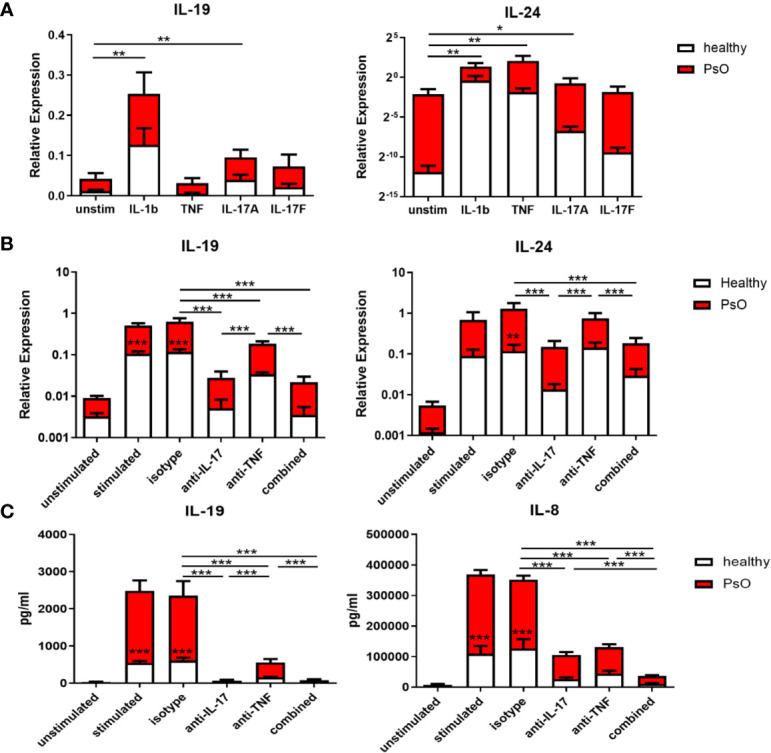
IL-17A induces IL-19 and IL-24 expression in human skin resident cells such as fibroblasts and keratinocytes, and IL-17A blockade reduces their expression. **(A)** IL-19 and IL-24 mRNA expression in fibroblasts of healthy donors (n=6) and psoriasis patients (n=4) without stimulation or stimulated with IL-1β, TNFα, IL-17A and IL-17F for 24 hours. **(B)** Healthy control skin fibroblasts and psoriatic fibroblasts (n=6 each) were co-cultured with unstimulated or anti-CD3/anti-CD28 stimulated memory CD4+ T cells from healthy controls for 72 hours. IL-19 and IL-24 mRNA expression in these co-cultures without treatment or treated with an anti-IL-17A antibody, anti-TNF antibody, their combination or an isotype control antibody. **(C)** Levels of human IL-19 and IL-8 protein in the supernatant of the above mentioned co-cultures without treatment or treated with an anti-IL-17A antibody, anti-TNF antibody, their combination or an isotype control antibody. Data are shown as means ± SEMs. *P < 0.05, **P < 0.01, and ***P < 0.001.

As the increase in IL-17 family cytokines preceded the upregulation of IL-19 and IL-24, we assumed that the IL-17 family cytokines induced IL-19 and IL-24. Therefore, the expression of IL-19 and IL-24 in skin fibroblasts was examined after stimulation with IL-1β, TNF, IL-17A, or IL-17F for 24 hours. The expression of the IL-17 receptor, IL-17RA and IL-17RC, was confirmed on skin fibroblasts from psoriasis patients or healthy volunteers (**Supplementary Figure S4A**). As shown in **Figure 5A**, both IL-17A and IL-1β significantly increased IL-19 and IL-24 mRNA expression compared to the unstimulated group. TNF, on the other hand, was only a significant inducer of IL-24 (**Figure 5A**). Additionally, we observed an intrinsic higher expression of IL-19 and IL-24 in psoriatic skin fibroblasts compared to skin fibroblasts from healthy controls (**Figure 5A**).

To further explore the induction of IL-19 and IL-24 by IL-17A, healthy and psoriasis skin fibroblasts were co-cultured with CD4+CD45RO+CD14-CD25low/int T memory cells for 72 hours with or without stimulation. Neutralizing antibodies for IL-17A, TNF or the combination were added to these co-cultures. **Figure 5B** showed that anti-IL-17A treatment significantly reduced mRNA expression of IL-19 and IL-24 compared to either the isotype antibody or anti-TNF. In contrast, anti-TNF treatment only significantly reduced expression of IL-19 but not of IL-24 (**Figure 5B**). No additive effect was shown when both IL-17A and TNF were neutralized compared to anti-IL-17 treatment alone. In line with the mRNA expression data, ELISA data showed that both anti-IL-17A and anti-TNF significantly reduced the protein levels of IL-19 in the co-culture supernatants with anti-IL-17 more potent than anti-TNF, but without additive effect when treatment was combined (**Figure 5C**). However, anti-IL17A and anti-TNF both significantly reduced the levels of IL-8, with additive effect when combined (**Figure 5C**). Also in these skin fibroblast-T cell co-cultures, IL-19 and IL-24 mRNA expression as well as protein levels of IL-19 and IL-8 were all lower when skin fibroblast were derived from healthy controls compared to patients with psoriasis (**Figures 5B, C**). This indicates an intrinsic higher expression of both cytokines in psoriasis lesions.

IL-17A-induced expression of IL-19 was also checked in healthy human skin primary keratinocytes. Expression of the IL-17 receptor, IL-17RA and IL-17RC, was confirmed in these keratinocytes (**Supplementary Figure S4A**). IL-17A stimulation of human keratinocytes resulted in increased IL-19 mRNA expression. On the other hand, inhibition of IL-17A in keratinocyte-T cell co-cultures reduced IL-19 mRNA expression (**Supplementary Figure S4B**). Overall, these data indicate that IL-17A induces IL-19 and IL-24 expression in human skin resident cells such as fibroblasts and keratinocytes, and IL-17A blockade reduces their expression.

### IL-17A Neutralization Reduces IL-19 and Cell Proliferation in Psoriatic Skin Lesions

**Figure 6:**
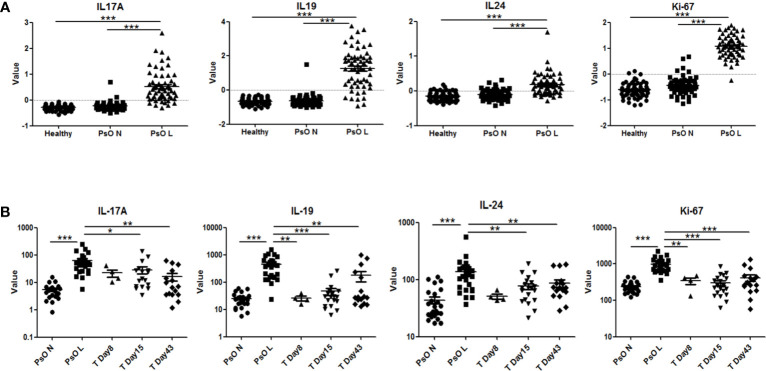
Expression of human IL-17A, IL-19, IL-24 and Ki-67 in psoriatic lesions and the effects of anti-IL-17RA therapy. **(A)** Expression of IL-17A, IL-19, IL-24 and Ki-67 in lesional and non-lesional skin biopsies from 58 psoriasis patients and in healthy skin biopsies from 64 normal controls in data set GSE13355. **(B)** Expression of IL-17A, IL-19, IL-24 and Ki-67 in lesional and non-lesional skin biopsies from 25 psoriasis patients before anti-IL-17RA treatment, 8, 15 and 43 days after treatments in data set GSE53552. Data are shown as means ± SEMs. *P < 0.05, **P < 0.01, and ***P < 0.001.

Gene expression data from psoriatic lesional skin, psoriatic non-lesional skin, and healthy skin, obtained in clinical trials with anti-IL-17 biologics in psoriasis, were analyzed using the Genomic Spatial Event (GSE) database. As shown in **Figure 6A**, IL-17A, IL-19, and IL-24 were all significantly higher in psoriatic lesional skin compared to either psoriatic non-lesional skin or healthy skin. This corroborated our findings of a higher expression of IL-19 and IL-24 in psoriatic skin fibroblasts compared to healthy fibroblasts (**Figure 5**). Furthermore, Ki-67, was significantly increased in psoriatic lesional keratinocytes (**Figure 6A**). Significant upregulation of IL-17A, IL-19, IL-24, and Ki-67 in psoriasis was confirmed in another independent dataset (**Supplementary Figure S5A**).

In a separate dataset, in which an IL-17 receptor A (IL-17RA) antibody was used in the treatment of psoriasis, a significant reduction of the expression of IL-19, IL-24, and Ki-67 in psoriatic lesional skin was found, reducing their expression levels close to those of non-lesional skin after treatment (**Figure 6B**). Similar significant down-regulation of IL-19 and Ki-67 was confirmed in another study using anti-IL-17A treatment (**Supplementary Figure S5B**). To summarize, multiple gene expression data of psoriatic patients before and after targeting the IL-17 pathway support our data that IL-17A regulates IL-19 expression in fibroblasts and keratinocytes.

## Discussion

In the present study, we showed that IL-10 neutralization enhanced skin inflammation, thickness and scaling in the IMQ-induced psoriasis mouse model beyond day 5, *via* upregulation of the IL-17/IL-19 axis. IL-17A induced IL-19 and IL-24 expression in human dermal fibroblasts and epidermal keratinocytes, and IL-17A neutralization reduced the expression of both cytokines. Gene array expression data also show high expression of IL-17A, IL-19, IL-24 and proliferation marker Ki-67 in psoriatic skin lesions, and that anti-IL-17 therapy reduced their expression. In addition to keratinocytes, dermal fibroblasts, through interaction with immune cells and cytokines such as IL-17A and TNF in psoriasis, can be a major source of IL-19 and IL-24 that contribute to perpetuation of psoriatic symptoms such as keratinocyte proliferation and acanthosis.

In psoriasis, recombinant human IL-10 treatment has been demonstrated to improve psoriatic symptoms in clinical trials (20–23). Similarly, in the IMQ-induced psoriasis mouse model, a subset of IL-10-producing B cells was identified, and adoptive transfer of these IL-10-producing B cells reduced disease severity (24). In contrast, as shown by our and other groups, IL-10 neutralization or IL-10 deficiency induced persistent psoriasis-like inflammation after IMQ application (25). In psoriatic skin, macrophages and DC both can be producers as well as direct target cells of IL-10 (26). Frequency of Th17 cells can also be directly controlled by IL-10 and vice versa (27, 28).

IL-10 belongs to the cytokine family of IL-19, IL-20, IL-22 and IL-24 and are located in the same cluster on chromosome 1 (29, 30). Although IL-10 is the only anti-inflammatory cytokine in this family, simultaneous induction of IL-10 and the IL-20 subfamily has been observed in monocytes by stimulants (31). IL-10 production during inflammation acts as a natural counter-balance to limit the side effects of inflammation. Without this Yin-Yang dynamic equilibrium, inflammation will be skewed towards uncontrolled harmful diseases. For instance, in psoriasis, low levels of IL-10 have been reported in comparison to other inflammatory skin conditions, while contrarily, enhanced expression of IL-23/IL-17 pathway cytokines has been widely confirmed in psoriatic lesions (9, 32, 33).

Overexpression of IL-19 and IL-24 has also been observed in psoriatic skin and both induce keratinocyte hyper-proliferation in a reconstituted human skin model (34–36), suggesting a pathogenic role in psoriasis. Myeloid cells are producers of both cytokines, and keratinocytes are also potential producers (29, 30, 37). Recently, IL-19 was suggested as an important mediator of the IL-23/IL-17 cascade in psoriasis, and IL-17A-induced expression of IL-19 in keratinocytes amplifies keratinocyte responses *via* auto-paracrine regulation (14). Here we confirmed the induction of IL-19 by IL-17A in human keratinocytes and extended to show that IL-17A neutralization reduced IL-19 in human keratinocyte-T cell co-cultures (**Supplementary Figure S4B**). In addition, our study provided evidence that dermal fibroblasts also produced IL-19 and IL-24 in response to IL-1β and IL-17A, and, when co-cultured with activated memory T cells, fibroblasts produced significant protein levels of IL-19 (**Figures 5A–C**). Synergistic induction of IL-20 subfamily cytokines by IL-1β and IL-17A has also been observed in recent publications (38). Interestingly, in psoriatic fibroblast-T cell co-cultures, higher levels of IL-19 was observed in comparison to healthy fibroblast co-cultures, further supporting the contribution of psoriatic fibroblasts to local IL-19 production. Compared to epidermal keratinocytes, dermal fibroblasts are positioned to encounter more frequently with inflammatory cells including T cells as most infiltrating T cells accumulate in the dermis (**Supplementary Figure S3A**). Therefore, in psoriasis, dermal fibroblasts could be an important local source of IL-20 subfamily cytokines and contribute to keratinocyte hyper-proliferation through a paracrine mechanism.

Discordant regulation of IL-20 in contrast to IL-19 and IL-24 has been found in our study. This could be due to different immune and resident cell types as potential cellular sources for these cytokines (30). Like skin fibroblasts and keratinocytes, other tissue cells such as endothelial cells and fibroblast-like synovial cells can also produce IL-20 subfamily cytokines (30, 39). Nevertheless, whether blocking the IL-20 subfamily cytokines will be viable options in psoriasis treatments still warrants further research.

In summary, we show that IL-10 regulates the expression of cytokines related to the IL-23/IL-17 axis *via* IL-19 and IL-24 influencing skin thickness and scaling. These data give further insight into the cytokine network in the stromal milieu of psoriasis plaques. Strategies to upregulate local or systemic production of IL-10 in patients with psoriasis could help increasing the effectiveness of current therapies.

## Data Availability Statement

The original contributions presented in the study are included in the article/**Supplementary Material**. Further inquiries can be directed to the corresponding author.

## Ethics Statement

The studies involving human participants were reviewed and approved by Medical Research Ethics Committee Erasmus MC. The patients/participants provided their written informed consent to participate in this study. The animal study was reviewed and approved by Erasmus MC Dutch Animal Ethics Committee (DEC).

## Author Contributions

XX contributed to the study design, performed experiments and wrote the manuscript. EF, PA, and A-MM performed experiments and revised the manuscript. PL and LB supported experiments and revised the manuscript. EP contributed to the study design and revised the manuscript. EL designed the study and revised the manuscript. All authors contributed to the article and approved the submitted version.

## Funding

The current project is funded by the Departments of Rheumatology and Dermatology, Erasmus MC, University Medical Center Rotterdam. XX is supported by a scholarship under China State Scholarship Fund (CSC No.201406100056).

## Conflict of Interest

The authors declare that the research was conducted in the absence of any commercial or financial relationships that could be construed as a potential conflict of interest.

## Publisher’s Note

All claims expressed in this article are solely those of the authors and do not necessarily represent those of their affiliated organizations, or those of the publisher, the editors and the reviewers. Any product that may be evaluated in this article, or claim that may be made by its manufacturer, is not guaranteed or endorsed by the publisher.
