# Supplementary material for: Interleukin-17A Drives IL-19 and IL-24 Expression in Skin Stromal Cells Regulating Keratinocyte Proliferation
Source: Front Immunol. 2021 Sep 20;12:719562. doi: 10.3389/fimmu.2021.719562 (PMC8488340; doi:10.3389/fimmu.2021.719562)
Supplement: Supplementary file 1 [file DataSheet_1.docx]

Supplementary Material

1. **Supplementary tables**

**Table S1. Primer sequences used in this study.**

| **Name** | **Species** | **Sequence** |
| --- | --- | --- |
| mIL-23p19 F | Mouse | CACCTCCCTACTAGGACTCAGC |
| mIL-23p19 R | Mouse | TGGGCATCTGTTGGGTCT |
| mIL-22 F | Mouse | TTTCCTGACCAAACTCAGCA |
| mIL-22 R | Mouse | CTGGATGTTCTGGTCGTCAC |
| mIL-17A F | Mouse | TTTTCAGCAAGGAATGTGGA |
| mIL-17A R | Mouse | TTCATTGTGGAGGGCAGAC |
| mIL17F F | Mouse | CAAGAAATCCTGGTCCTTCG |
| mIL17F R | Mouse | GAGCATCTTCTCCAACCTGAA |
| mIL-19 F | Mouse | TGGAGAACCTCAGGAGCATT |
| mIL-19 R | Mouse | GAATGTCAGCAGGTTGTTGG |
| mIL-24 F | Mouse | AGAACCAGCCACCTTCACAC |
| mIL-24 R | Mouse | GTGTTGAAGAAAGGGCCAGT |
| mCXCL2 F | Mouse | AAAATCATCCAAAAGATACTGAACAA |
| mCXCL2 R | Mouse | CTTTGGTTCTTCCGTTGAGG |
| mKC (CXCL1) F | Mouse | GACTCCAGCCACACTCCAAC |
| mKC (CXCL1) R | Mouse | TGACAGCGCAGCTCATTG |
| mGAPDH F | Mouse | AGCTTGTCATCAACGGGAAG |
| mGAPDH R | Mouse | TTTGATGTTAGTGGGGTCTCG |
| IL-19 F | Human | GGAGACTCTGCAGATCATTAAGC |
| IL-19 R | Human | GATCCTTGAACACCCTGTCC |
| IL-24 F | Human | GAAGAATTGAGGCTGCTTGG |
| IL-24 R | Human | GAGGGCAGAAGGGTCTGG |
| HPRT F | Human | TGACCTTGATTTATTTTGCATACC |
| HPRT R | Human | CGAGCAAGACGTTCAGTCCT |

1. **Supplementary Figures**

**Figure S1. Experimental design of anti-IL-10 therapy in the IMQ-induced psoriasis mouse model.** (A) Expression of IL-10 in lesional skin after topical Aldara application in the IMQ-induced mouse model at day 5. (B) Experimental design and groups of the anti-IL-10 treatment experiments in IMQ-induced psoriasis mouse model sacrificing mice at days 5 and 10. Data are shown as means ± SEMs. ** *P*<0.01.

**Figure S2. Kinetic information of macroscopic and histologic scores and infiltration of myeloid cells after anti-IL-10 treatment in the IMQ-induced mouse model.** (A) Time-course follow-up of local PASI scores, including skin scaling, thickness and redness, in anti-IL-10, isotype control antibody and dexamethasone-treated groups during 10 days of Aldara application. (B) Summary of days 5 and 10 epidermal thickness and thickness ratios in all groups including saline-treated IMQ group. (C-D) Summary of percentages and cell numbers of neutrophils (C) and monocyte-derived dendritic cells (D) in about 1 cm^2^ lesional digested skin from all groups including saline-treated IMQ group at day10. Data are shown as means ± SEMs. * *P*<0.05, ** *P*<0.01, and *** *P*<0.001.

**Figure S3. T cell subsets infiltration in IMQ-applied skin after IL-10 neutralization compared to isotype antibody group.** (A) Representative CD3+ IHC staining in anti-IL-10 and isotype antibody groups 10 days after IMQ application. (B) Representative flow cytometry staining of CD3+ T cells among CD45+ immune cells in anti-IL-10 and isotype antibody groups 10 days after IMQ application. (C) Cell number summary of infiltrating CD3+ T cells, CD3+CD4+ T cells, and CD3+TCRγδ+ T cells in anti-IL-10 and isotype antibody groups (n=6 each) 10 days after IMQ application.

**Figure S4. Fibroblasts and keratinocytes express IL-17RA and IL-17RC and respond to IL-17A stimulation.** (A) Representative staining of IL-17RA and IL-17RC compared to isotype antibody controls on healthy and psoriatic skin fibroblasts, as well as healthy skin keratinocytes. (B) mRNA expression of IL-19 in keratinocytes stimulated with IL-17A for 24 hrs (left panel) and in anti-CD3/anti-CD28 stimulated co-cultures of keratinocytes and sorted healthy CD4+ T memory cells in which neutralizing antibodies against IL-17A, TNF, the combination of these antibodies or an isotype control antibody was added (right panel).

**Figure S5. Expression of IL-19, IL-24 and Ki-67 in psoriatic lesions and the effect of anti-IL-17A antibody treatment.** (A) Expression of IL-17A, IL-19, IL-24 and Ki-67 in lesional (PsO L) and non-lesional (PsO N) skin biopsies from 4 psoriasis patients in dataset GSE50790. (B) Normalization of IL-19, IL-24 and Ki-67 in lesional biopsies from psoriasis patients before (aIL-17 w0, n=8) and after (aIL-17 w2, n=6) 2-weeks anti-IL-17A treatment with placebo (n=8 each) treatment controls in dataset GSE31652. Data are shown as means ± SEMs. * *P*<0.05, ** *P*<0.01, and *** *P*<0.001.
